# Impaired Decisional Impulsivity in Pathological Videogamers

**DOI:** 10.1371/journal.pone.0075914

**Published:** 2013-10-16

**Authors:** Michael A. Irvine, Yulia Worbe, Sorcha Bolton, Neil A. Harrison, Edward T. Bullmore, Valerie Voon

**Affiliations:** 1 Department of Psychiatry, University of Cambridge, Cambridge, United Kingdom; 2 Behavioural and Clinical Neuroscience Institute, University of Cambridge, Cambridge, United Kingdom; 3 Cambridgeshire and Peterborough NHS Foundation Trust, Cambridge, United Kingdom; 4 Brighton and Sussex Medical School, University of Sussex, Brighton, United Kingdom; Institute of Psychiatry at the Federal University of Rio de Janeiro, Brazil

## Abstract

**Background:**

Pathological gaming is an emerging and poorly understood problem. Impulsivity is commonly impaired in disorders of behavioural and substance addiction, hence we sought to systematically investigate the different subtypes of decisional and motor impulsivity in a well-defined pathological gaming cohort.

**Methods:**

Fifty-two pathological gaming subjects and age-, gender- and IQ-matched healthy volunteers were tested on decisional impulsivity (Information Sampling Task testing reflection impulsivity and delay discounting questionnaire testing impulsive choice), and motor impulsivity (Stop Signal Task testing motor response inhibition, and the premature responding task). We used stringent diagnostic criteria highlighting functional impairment.

**Results:**

In the Information Sampling Task, pathological gaming participants sampled less evidence prior to making a decision and scored fewer points compared with healthy volunteers. Gaming severity was also negatively correlated with evidence gathered and positively correlated with sampling error and points acquired. In the delay discounting task, pathological gamers made more impulsive choices, preferring smaller immediate over larger delayed rewards. Pathological gamers made more premature responses related to comorbid nicotine use. Greater number of hours played also correlated with a Motivational Index. Greater frequency of role playing games was associated with impaired motor response inhibition and strategy games with faster Go reaction time.

**Conclusions:**

We show that pathological gaming is associated with impaired decisional impulsivity with negative consequences in task performance. Decisional impulsivity may be a potential target in therapeutic management.

## Introduction

Pathological gaming in adolescents and young adults is an emerging problem in developed societies with the rapid escalation of technological advances. The frequency has been reported between 7.5% to 11.9% in various countries (reviewed in [1]). A recent meta-analysis of published studies focusing on the stringent criterion of interference of functioning documented a frequency of 3.1% [2] suggesting the problem is common. The pathological form of the behaviour predicts poorer functioning and is associated with greater depression and anxiety, poorer school performance and impaired social interaction [1], [2]. The proposed revision of the Diagnostic and Statistical Manual of Mental Disorders (DSM), Version 5, will likely include internet use disorder in Section III as a condition requiring further research, of which pathological gaming is a subset [3]. Many studies use screening tools adapted from DSM IV-TR diagnostic criteria from established substance or behavioural addictions such as pathological gambling to assess gaming severity [1], [4]–[11].

There is some evidence that pathological gaming might have overlaps with other behavioural and substance addictions. For instance, playing a video game in healthy volunteers is associated with greater ventral striatal presynaptic dopamine release suggesting that the act of video game-playing is potentially rewarding or motivating in itself [12]. Healthy adolescents with frequent video game playing had greater left striatal grey matter volume, a region that also had greater activity during loss feedback and correlated negatively with deliberation time on the Cambridge Gamble Task [13]. Following a 6 week extended gaming exposure in healthy volunteers, gaming cues increased orbitofrontal and anterior cingulate activity suggesting that the act of video game-playing can be reinforcing and associated cues can become conditioned reinforcers [14]. Similarly, subjects with pathological gaming have a greater cognitive bias and cue reactivity towards game-related images with greater medial prefrontal and anterior cingulate cortex activity [11], [15], [16]. In subjects with pathological gaming, an 18FDG PET imaging study demonstrated greater glucose metabolism in the orbitofrontal cortex, caudate and insula and decreased metabolism in sensorimotor and occipital cortices [17]. Pathological gaming is also associated with greater impulsivity on the Barratt's Impulsiveness Scale and greater perseveration on the Wisconsin Card Sorting Test along with increased volume in thalamus and inferior temporal and occipital gyri [18]. Pathological gamers further demonstrated impaired motor response inhibition on a motor response inhibition task, the Go/No Go task, along with decreased error-related negativity [19]. Together these studies suggest potential overlapping mechanisms with other behavioural and substance addictions.

Impulsivity is heterogeneous, divisible into decision and motor impulsivity, and is commonly impaired in substance and behavioural addictions [20], [21]. Decision impulsivity includes impulsive choice, or a preference for an immediate smaller reward over a larger delayed reward, which can be tested using delay discounting tasks [22], and reflection impulsivity or the tendency to gather and appraise information prior to making a decision [23], which can be tested using the Information Sampling Task. Motor impulsivity includes motor response inhibition which can be tested using the Stop Signal Task (SST) [24] which assesses action cancellation and the Go/No Go task which assesses action restraint. Motor impulsivity also includes premature responding, or anticipatory responding which can be tested using a novel translational task in humans [25]. In rodent studies, impulsivity characterized by premature responding and delay discounting are endophenotypic predictors of the development of substance use disorders [20], [26], [27].

Here we investigated decisional and motor impulsivity using four measures of impulsivity in subjects with pathological video game use (VG). We focused on the diagnosis of VG rather than the broader issue of internet addiction. We hypothesized that VG would be associated with greater decision impulsivity, both in terms of delay discounting and reflection impulsivity. Although a previous study has shown an impairment in motor response inhibition with the Go/No Go task which involves both action selection and action restraint, we focused on the SST, which measures action cancellation. Video game use can have clear beneficial effects in healthy volunteers with reports of greater visual acuity as indexed by contrast sensitivity [28], improved attentional flexibility [29], and improved reaction times [30]. In a study of internet addiction in which 71% were involved in online gaming, those with internet addiction had improved decision-making on the Iowa gambling task, and no impairments in the Balloon Analogue Risk Test (BART) of risk-taking [31]. Although we predicted an impairment in decisional impulsivity, we did not predict an impairment in measures of motor impulsivity as their performance might be mitigated by practice effects from video game use. Along these lines, we further explored the relationship between game subtypes and task outcomes.

## Materials and Methods

### Ethics Statement

The study was approved by the University of Cambridge Research Ethics Committee. All subjects provided written informed consent.

### Recruitment

Subjects were recruited via community- and university-based advertisements in Cambridge. Subjects were included if they were 18 or older. In keeping with other published studies, VG was diagnosed based on a 10 item adaptation of the DSM IV pathological gambling criteria [1], [4]–[6], [8], [32]–[37]. The relative frequency with which this approach is taken is illustrated by a recent review of psychometric assessment tools [38].

Subjects were excluded if they had a current major depressive episode or a history of a severe psychiatric disorder (e.g. bipolar affective disorder or schizophrenia) or a current substance use disorder including regular cannabis use. All diagnoses were reviewed by a psychiatrist. Healthy volunteers were excluded if they were regular nicotine users. Subjects were excluded if they tested positive for a drug urine screen (including cannabis) or alcohol breathalyzer test on the day of testing.

### Procedure

After providing written consent all subjects underwent urine drug testing and an alcohol breathalyzer test on the day of testing. Subjects completed the Beck Depression Inventory-II [39] to assess depressive symptoms and the UPPS Impulsive Behaviour Scale to assess impulsivity [40]. VG severity was also assessed with the well-established Game Addiction Scale (GAS; 7-item version) [10]. A version of the Yale-Brown Obsessive Compulsive Scale (YBOCS) [41]) was adapted specifically for videogaming to assess for VG severity similar to the YBOCS adaptation for gambling [42]. We also quantified the frequency or number of days played in role playing games (multiplayer online role-playing games and single player role playing games), strategy-type games (strategy, puzzle) and in reaction time games (sports, first person shooter, platform and racing games) (Scoring: 6 =  Nearly every day; 5 = 3–5 days/week; 4 = 1–2 days/week; 3 = 2/3 days per month; 2 =  less often; 1 =  never). The scores were averaged for the role playing, strategy-type and reaction time games. Subjects were screened for comorbid psychiatric disorders with the Mini International Neuropsychiatric Inventory (MINI; [43]). The National Adult Reading Test (NART; [44]) was used to obtain indices of premorbid IQ. Subjects were remunerated at a rate of £7.50 per hour including travel costs, with an additional £5 contingent on task performance.

### Information Sampling Task (IST)

The IST is a task from the Cambridge Neuropsychological Test Automated Battery (CANTAB) [23]. Subjects viewed a 5×5 matrix of grey boxes on a touch screen monitor. Upon being touched, boxes opened to reveal one of two colours. The objective was to decide which of the two colours was predominant in the matrix, by opening a sufficient number of boxes in order to be able to make that decision. In the No Cost condition, subjects could win 100 points for correct choices or lose 100 points for incorrect choices regardless of the number of boxes opened. In the Cost condition, the possible number of points for a correct answer started at 250, and decreased by 10 with every box opened. Thus subjects could win more points for earlier decisions. The penalty for a wrong answer remained the same at 100 points. Once subjects had made a decision they touched the corresponding coloured panel below the matrix. A message appeared for 2 seconds – “Correct! You have won [x] points” or “Wrong! You have lost 100 points”. There were 10 self-paced trials for each condition. An inter-trial interval (minimum 1 second) was adjusted so that each trial lasted at least 30 seconds to counteract delay-averse responding. The primary outcome measure was the average number of boxes opened. Secondary measures included total points, sampling errors (incorrect choices) and probability correct (probability that the subject is correct at the time of decision).

### Delay Discounting Task

Delay discounting refers to the tendency to discount delayed rewards and is commonly measured using the Monetary Choice Questionnaire [22]. The questionnaire is a 27-item, self-administered questionnaire in which participants choose between a small immediate reward, and a larger delayed reward (e.g. Would you prefer £14 today, or £25 in 19 days?). The primary outcome measure was the slope (k) of the discounting curve calculated as follows: V = A/(1+ kD) where V is the present value of delayed reward A at delay D. The higher the k value, the steeper the slope and the greater the discounting or impulsive choice. The k value of small, medium and large magnitude choices were averaged for the final k value.

### Premature responding task

The premature responding task is a novel translation of the rodent 5-choice serial reaction time task [25]. Subjects pressed and held down the space bar with their dominant index finger when 4 boxes appeared on a touch screen monitor. The space bar press indicated the ‘cue onset’ time. A green circle target appeared briefly (32 to 64 ms) and randomly in one of the boxes after a specified time (cue-target interval: 2 to 10 seconds). Subjects released the space bar and touched the box on the screen in which the target had appeared, and were told that the amount of money earned was dependent on how quickly they responded. There were 2 Baseline blocks without monetary feedback and 4 Test blocks with monetary feedback. Mean reaction time (RT) in the Baseline blocks was used in order to set individualized monetary feedback (20 trials per block; at the start and after the first Test block) to encourage faster responding. In the Test blocks, subjects won more money for faster responses and lost money for late responses. Following premature or incorrect responses, subjects touched the screen to complete the trial, which was followed by a ‘Keep going’. The Test Blocks varied by target duration, cue-target interval and the presence of distractors. The primary outcome measures were premature release of the space bar prior to onset of the target and a Motivation Index  =  (Baseline RT1 – Baseline RT2)/(Baseline RT1+ Baseline RT2). The Motivation Index measured RT in extinction without feedback following instrumental learning with feedback with a higher score represented greater motivation [25].

### Stop Signal Task (SST)

The SST is also a task from the CANTAB [24]. Subjects viewed a computer screen and responded on a two button response box using both index fingers. Subjects pressed the right or left button for a “Go” stimulus (arrow appearing within a circle pointing either left or right) which remained on screen until the subject responded. In 20% of the trials, they are required to withhold any response when an audible “beep” is sounded (Stop signal). The Stop signal occurred 250 milliseconds after the Go signal (Stop Signal Delay, SSD). The SSD varied in a step-wise manner dependent on the previous response, decreasing by 50 milliseconds for a successful stop and increasing by 50 milliseconds for unsuccessful stops. Thus, successful stopping occurred at approximately 50% of the trials. The task had 5 blocks of 80 trials. The primary outcome measure was the stop signal reaction time (SSRT), which was calculated as follows: SSRT  =  median Go reaction time – SSD [45] in which a higher score indicated greater impairment in response inhibition.

### Statistics

The data was inspected for outliers and normality of distribution tested using Shapiro-Wilkes test. The subject characteristics and the 4 primary outcome measures (IST boxes opened, DDT, SSRT, premature responding) were analysed using an independent t-test. For the IST, secondary analyses were conducted to assess the total points and errors and the effects of cost. A mixed model ANOVA was used to measure the primary outcome measure of evidence sampled (boxes opened) with Group (VG, healthy volunteers) as a between-subjects factor and Cost (No cost, cost) as a within-subjects factor. Similar mixed model ANOVAs were also used to assess total points and sampling error. In order to control for the effects of nicotine use, all analyses were repeated with nicotine use as a covariate. Correlational analyses were conducted between measures of VG severity and task outcomes using Pearson correlation. Linear regression using the forward method to assess for independent variables and control for multiple comparisons was used to assess the relationship between types of games played and task outcomes. The variables of IST boxes opened, DDT, GoRT, SSRT, premature responding and Motivational Index were entered into the model. P<0.05 was considered significant.

## Results

Twenty-six VG subjects (23 males, mean age 24.69 (S.D. 5.90) years, Verbal IQ 119.80 (SD 4.33)) were compared with twenty-six age-, gender- and IQ-matched healthy volunteers (23 males, mean age 25.61 (SD 5.87) years, Verbal IQ 118.13 (SD 4.58)) There were no differences in mean age (d.f = 50, t = 0.56, p = 0.57) or IQ (d.f = 50, t = 1.35, p = 0.18). Seven VG subjects were nicotine users. The severity of pathological gaming is given in Table 1. There were no differences in subject characteristics, UPPS or BDI scores (Table 2).

**Table 1:** Videogame-playing measures.

Measure	Mean (+/− S.E)	Range
No. diagnostic criteria	7.29 (0.40)	5–10
Hours played per day	5.2 (0.37)	3.5–11
Days played per week	6.4 (0.13)	5–7
Hours played per week	33.29 (3.03)	21–84
YBOCS-G score	15.42 (0.70)	12–25
GAS score	4.93 (0.23)	2–7
Role playing games	2.37 (0.28)	1–6
Strategy games	2.37 (0.25)	1–6
Reaction time games	1.74 (0.19)	1–5

Abbreviations: SE  =  standard error; YBOCS-G  =  modified Yale Brown Obsessive Compulsive Score adapted for video gaming.

**Table 2:** Outcome measures (data are mean +/− S.D).

Instrument/task	Measure	HV	VG	T	P-value
UPPS		128.37 (20.93)	136.79 (29.60)	1.56	0.12
BDI		4.29 (5.03)	7.61 (5.28)	2.02	0.05
DDT	K-value	0.02 (0.04)	0.07 (0.1)	2.86	0.006
IST	Boxes opened	17.89 (5.72)	14.13 (5.74)	2.07	0.04
Premature responding	Premature responding	6.82 (5.29)	10.36 (9.12)	1.71	0.09
	Motivational Index	0.13 (0.16)	0.18 (0.21)	−0.97	0.34
Response inhibition	GoRT	367.22 (81.04)	366.27 (100.23)	0.04	0.97
	SSRT	160.81 (49.95)	154.27 (31.49)	0.52	0.60

Abbreviations: HV  =  healthy volunteers; VG  =  pathological gamers; UPPS  =  UPPS Impulsive Behaviour Scale; BDI  =  Beck Depression Inventory-II; DDT  =  Delay Discounting Task; IST  =  Information Sampling Task.

**Figure 1:**
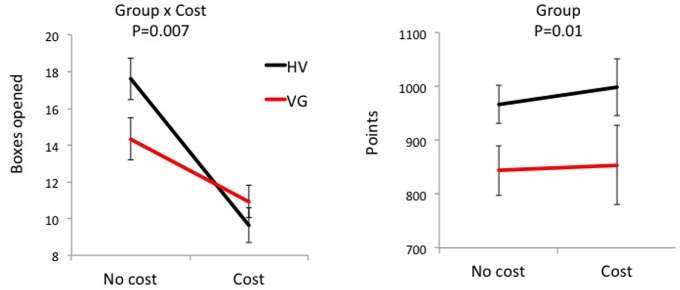
Information sampling task outcome measures. Mixed measures ANOVA of Information Sampling Task outcomes as a function of cost. Left: Boxes opened; Right: Points. Abbreviations: HV  =  healthy volunteers; VG  =  pathological gamers; YBOCS-G  =  modified Yale-Brown Obsessive Compulsive Score for gaming.

For the primary outcome measure of the IST, VG subjects sampled less evidence (boxes opened) in the No Cost condition compared to healthy volunteers (p = 0.04; Figure 1). Secondary analyses were used to assess the effects of Cost and the measures of total points and errors. There was a main effect of Cost on evidence sampled (F(1,50)  = 50.47, P<0.0001) but no main effect of Group (F(1,50)  = 0.70, P = 0.41). There was a Group by Cost interaction (F(1,50)  = 8.00, p = 0.007) in which VG subjects opened fewer boxes in the No Cost condition compared to HV (mean difference  = 3.26 (95% CI = 0.09–6.42), F(1,50)  = 4.27, p = 0.04) with no difference in the Cost condition (mean difference  = −1.28 (95% CI = −3.81–1.25), F(1,50)  = 1.03, p = 0.32) (Figure 1). The Group by Cost interaction remained significant including with nicotine use as a covariate (F(1,49)  = 5.64, p = 0.02).

In the IST total points, there was also a main effect of Group (F(1,50)  = 6.98, p = 0.01) in which VG scored fewer total points. There was no effect of Cost (F(1,50)  = 0.15, p = 0.70) or an interaction effect (F(1,50)  = 0.04, p = 0.84. The main Group effect remained significant with nicotine use as a covariate (F(1,49)  = 4.01, p<0.05). In the IST sampling errors, there was an effect of Cost (F(1,50)  = 16.73, p<0.0001) but no effects of Group (F(1,50) <0.0001, p = 0.99) or interaction (F(1,50)  = 2.22, p = 0.14). In the IST probability correct, there was an effect of Cost (F(1,50)  = 28.48, p<0.001) but no effects of Group (F(1,50)  = 0.46, p = 0.50) or interaction effect (F(1,50)  = 2.40, p = 0.13).

**Figure 2:**
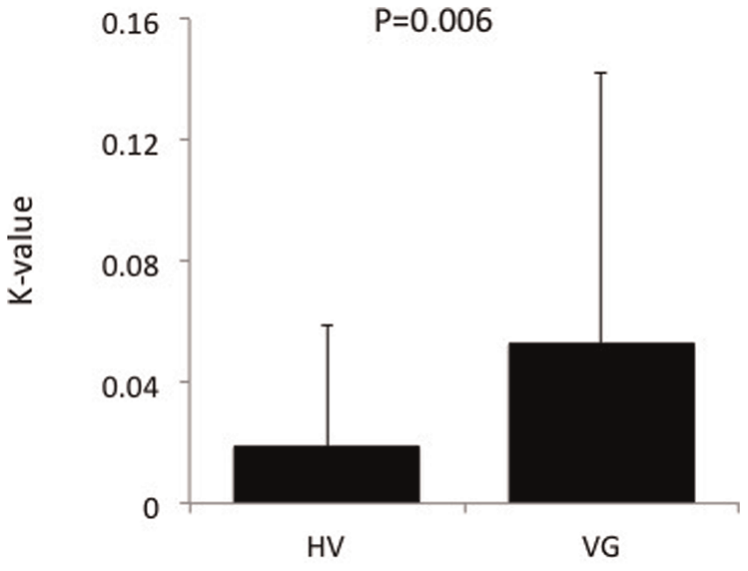
Delay discounting and information sampling task. K-value of the delay discounting task.

For the DDT, 1 healthy volunteer and 3 VG subjects were excluded from the analysis as outliers (>3 SD above mean). VG subjects made more impulsive choices relative to HV (Table 2 and Figure 2) (p = 0.006). With nicotine use included as a covariate, the difference between groups remained significant (F = 3.38, p<0.05).

There was a trend towards greater premature responding in VG compared to healthy volunteers (p = 0.09) (Table 2). However, the group difference was not significant with nicotine as a covariate (F(1,49)  = 2.10, p = 0.15). There were no group differences in SSRT (p = 0.60) (Table 2).

**Figure 3:**
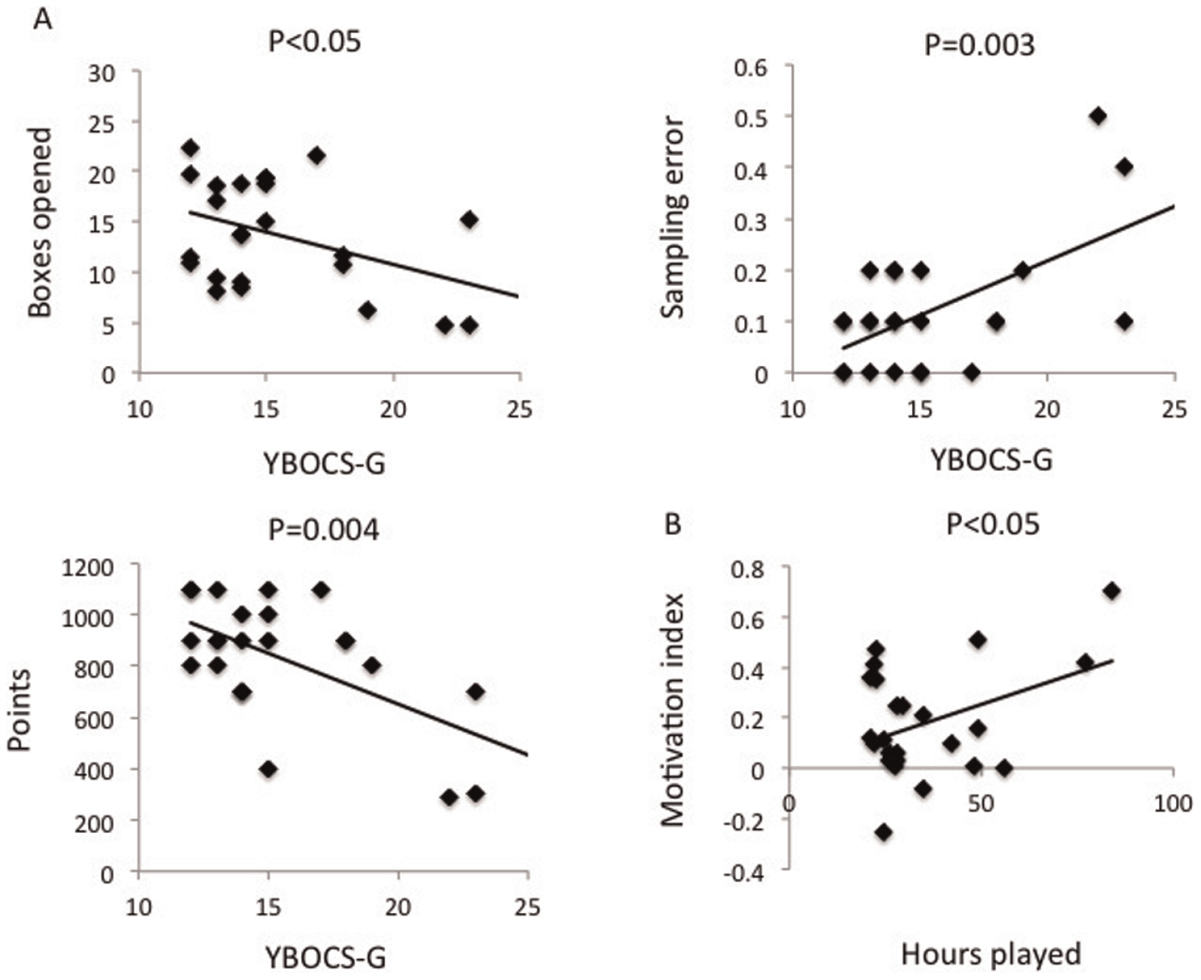
Correlation of severity measures with task outcomes. A. Correlation analyses of video gaming severity based on modified Yale Brown Obsessive Compulsive Scale scores adapted for gambling with Information Sampling Task outcome measures. B. Correlation analysis of video game hours played per week with Motivation Index from the premature responding task.

There was a negative correlation between the severity of gaming as measured using YBOCS-VG and boxes opened in the No Cost condition (reported as Pearson correlation coefficient: r = −0.41, p<0.05) (Figure 3) and with points scored (r = −0.57, p = 0.004) along with a positive correlation with sampling error (r = 0.58, p = 0.003). The number of hours played per week was positively correlated with Motivational Index (r = 0.40, p<0.05) (Figure 3). There were no correlations with the GAS score (p>0.05).

**Figure 4:**
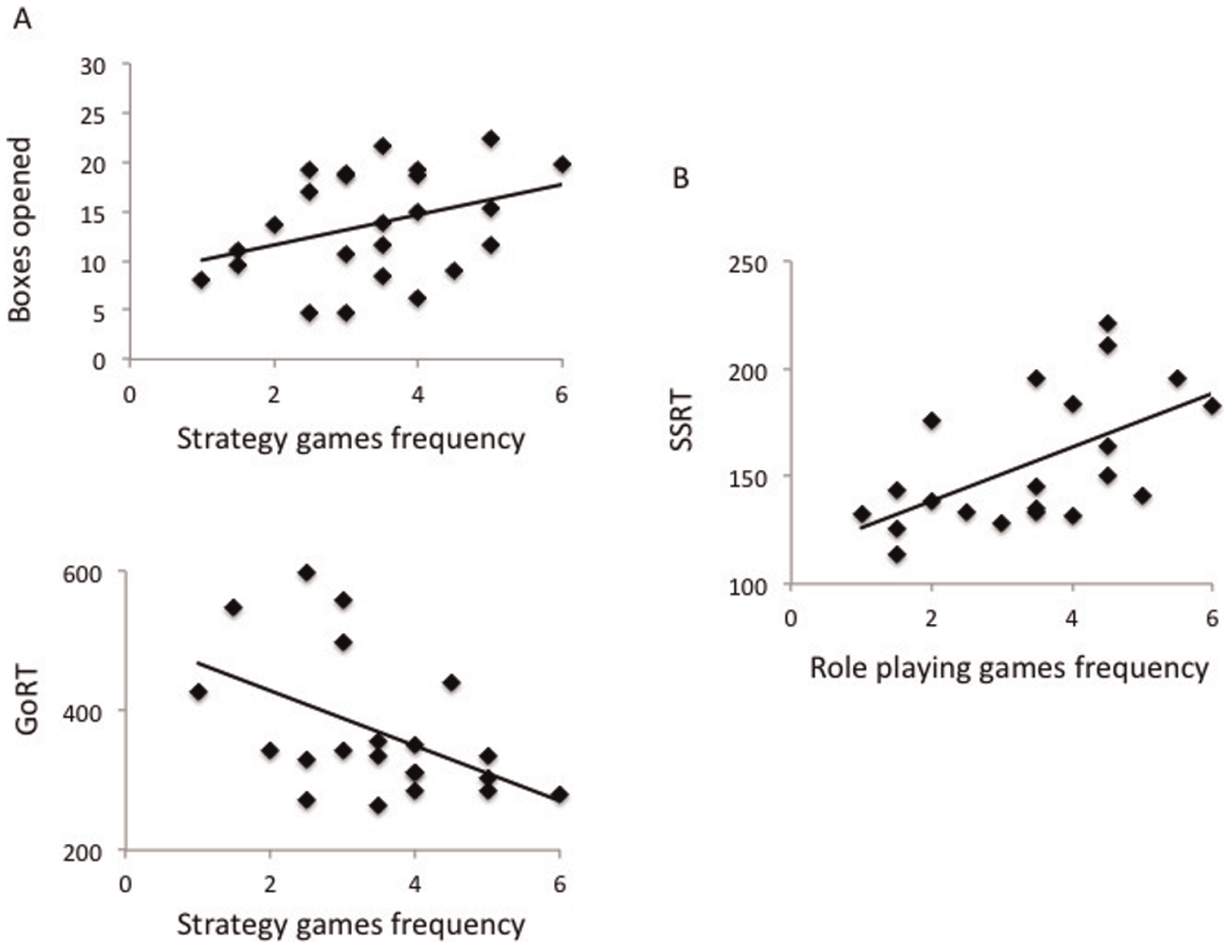
Linear regression of game types with task outcomes. A. Linear regression analysis of the frequency of strategy games played (p = 0.02) with Go Reaction Time (GoRT) from the Stop Signal Task and boxes opened from the Information Sampling Task remaining in the model. B. Linear regression analysis of the frequency of role playing games played (p = 0.01) with Stop Signal Reaction Time (SSRT) remaining in the model.

Using linear regression analysis, greater frequency of role playing games was associated with impaired motor response inhibition (higher SSRT) (R^2^ = 0.31, p = 0.01) with SSRT as an independent factor identified in the model (t = −2.85, p = 0.01) (Figure 4). Greater frequency of strategy games was associated with faster GoRT on the SST task and decreased reflection impulsivity (more boxes opened on the IST) (R^2^ = 0.39, p = 0.02) with faster GoRT (t = 2.58, p = 0.02) and boxes opened (t = −2.10, p = 0.051) identified as independent factors in the model (Figure 4). Frequency of reaction time games was not associated with any significant variables in the model.

In both groups and each group separately, there was no relationship between the outcome variables of IST boxes opened, SSRT, DDT, or premature responding (Pearson correlation coefficient r = −0.18–0.29, p<0.05).

## Discussion

We show that pathological gaming is associated with greater decision impulsivity, with less evidence sampled prior to a decision and greater impulsive choice. Greater reflection impulsivity (i.e. sampling less evidence or opening fewer boxes prior to making a decision), had negative consequences with pathological gamers obtaining significantly fewer points, possibly mediated via the numeric increase in number of sampling errors. This effect might be driven by subjects with greater gaming severity as greater YBOCS-VG scores were positively correlated with less evidence sampled, more sampling errors and fewer points acquired.

We further show that the introduction of a cost or penalty to the amount of evidence sampled is associated with less of a decrease in evidence sampled in pathological gamers relative to controls. Whereas both groups decrease the amount of evidence sampled with cost as expected, pathological gamers have significantly less of a decrease. In the No Cost condition, the optimal strategy to increase points is to sample as much evidence as possible. In contrast, in the Cost condition, each increment of evidence sampled is associated with loss of points. Despite an improvement in reflection impulsivity in the Cost condition in the pathological gamers, since the greater evidence sampled was associated with greater penalties, overall pathological gamers still obtained fewer points compared to the healthy volunteers. There were no significant group or interaction differences in sampling errors (incorrect choices) or probability correct (likelihood the subject is correct at the time of decision) suggesting the loss of points was not driven by errors but by points lost due to greater evidence sampled. Overall our findings suggest that pathological gamers might be less sensitive to the introduction of cost or penalties or may be more impaired in the integration of decision cost in optimizing final outcomes.

In contrast, the number of hours played was positively correlated with a Motivational index in the premature responding task. This measure of motivation assesses reaction time in extinction following instrumental conditioning with monetary feedback (24) suggesting that motivation in pathological gamers may be influenced by instrumental rewarding feedback. Thus, pathological gaming subjects responded faster to the target when tested in extinction without feedback, after the subjects have learned that responding to the target can lead to a reward feedback. This data along with the Information Sampling Task data suggests that negative cost on decisional choices may be less effective than rewarding feedback to modify behaviours in pathological gamers.

Greater impulsivity is commonly observed in substance use disorders and in behavioural addictions such as pathological gambling. Decision impulsivity, such as impulsive choice, and reflection impulsivity, as measured in this current study, is commonly impaired across a range of substance use disorders. An extensive literature pertains to an association between elevated reflection impulsivity and impulsive choice and various substance use disorders, including opiates [23], stimulants [46] and alcohol [47], [48]. Similarly, pathological gambling is also associated with elevated impulsivity [47]. In this current study, we excluded subjects with concomitant substance use (including cannabis) and controlled for comorbid nicotine use suggesting the results are unrelated to comorbid substance use.

Whether these impairments are predictive traits and shift an individual towards pathological behaviour or are state-specific and related to excessive gaming is not known. We observed a dissociation between group outcomes in motor and decision impulsivity measures. Unlike impairments in motor impulsivity commonly observed in substance use disorders in measures of response inhibition as measured using the SST [49] and premature responding [25], no differences in motor impulsivity were observed in VG subjects. These measures are commonly impaired in substance use disorders both as a state and trait effect. This contrasts with the report of greater impairments in motor inhibition in VG subjects using the Go/No Go task [19] which assesses both the selection of an action and action restraint rather than action cancellation assessed using the SST. That greater engagement in role playing games is associated with impairments in SST suggests that motor response inhibition is possibly impaired in those that focus on role playing games. Thus, that we did not find response inhibition differences in the SST as compared to the observation of impairments in the Go/NoGo task might reflect task differences or population differences in the types of games played. Pathological gaming subjects had a trend towards greater premature responding, which was explained by concomitant nicotine use. This lack of group difference in motor impulsivity might also suggest either that the excessive gaming does not worsen motor control or possibly improves motor control if there is a baseline impairment. The lack of an effect might also suggest potential differences between pathological gaming and disorders of substance use.

We further investigated the influence of type of game played by separating out reaction time (‘fast twitch’) games, strategy games and role playing games, the latter of which may have elements of both strategy (character development and statistics) and action sequences. We demonstrated that a greater frequency of role playing games was associated with impaired motor response inhibition and a greater frequency of strategy games with less reflection impulsivity (more evidence sampled in the IST) and faster GoRT in the SST. We did not observe any significant associations with reaction time games. This data argues against a role for excessive video gaming in improving motor control in pathological gamers in which one might expect that games focusing on reaction time and motor control and less on strategy might be associated with a faster reaction time and improved motor response inhibition.

There were several limitations in the current study. The number of subjects may not be sufficiently large to fully document group differences. However, this is predominantly an issue for trend or negative findings. Although there is no unanimous consensus on diagnostic criteria for pathological gaming, we used stringent criteria focusing on functional impairment and based criteria on pathological gambling, a well-validated behavioural addiction whose diagnostic criteria have previously been adapted for this purpose [9], [10]. We also controlled carefully for comorbid substance use although differences as a result of comorbid substance use may also be very instructive. We studied subjects who were not currently treatment-seeking suggesting possibly a milder form. However, we still demonstrate clear abnormalities. Future studies may further investigate the types of games played. This would allow for inferences about the relationship between motor impulsivity, reaction times and practice effects.

We show that pathological gamers have impaired decisional forms of impulsivity. Unlike studies in healthy volunteers with excessive non-pathological gaming, we did not demonstrate any improvements in cognitive or motor measures. This study further contributes to our understanding of this behaviour and particularly highlights impairments in impulsivity in the pathological forms of video gaming. Our data suggests pathological gamers may be more likely to respond to instrumental reward feedback and less to negative costs in decision making. These impairments may also represent possible therapeutic targets for cognitive therapy in the management of pathological gaming.
